# Selective-wavelength perfect infrared absorption in Ag@ZnO conical metamaterial structure

**DOI:** 10.1038/s41598-024-71260-2

**Published:** 2024-09-12

**Authors:** Muhammad Faisal, Atta Ur Rahman, Sajid Khan, Muhammad Siyaf, Tawaf Ali Shah, Mohammad K. Okla, Mohammed Bourhia, Youssouf Ali Younous

**Affiliations:** 1 Department of Physics, Kohat University of Science and Technology, Kohat, KPK Pakistan; 2 Department of Physics, Khushal Khan Khattak University, Karak, KPK Pakistan; 3 College of Agriculture Engineering and Food Sciences, Shandong University of Technology, Zibo, 25500 China; 4 Botany and Microbiology Department, College of Science, King Saud University, P.O. Box, Riyadh, Saudi Arabia; 5 Department of Chemistry and Biochemistry, Faculty of Medicine and Pharmacy, Ibn Zohr University, 70000 Laayoune, Morocco; 6 Evangelical College, BP 1200, N’Djamena, Chad

**Keywords:** Metamaterial perfect absorber, Surface plasmon polariton, Magnetic polariton, IR stealth technology, Materials science, Physics

## Abstract

We present a new selective Metamaterial Perfect Absorber (MPA) consisting of zinc oxide embedded silver (Ag@ZnO), designed for applications in infrared stealth technology. The numerical simulation included a wide frequency range from 1 to 1000 THz and shows that the design MPA structure presented two absorption peaks at the desired wavelengths of 1.7 µm and 6.5 µm. The absorptivity of both peaks reached approximately 93.1% and 93.5%. The first peak at 1.7 µm decreases the scattering of IR laser beams from the surface of the MPA structure and also lowers the infrared tracks that could direct laser-guided devices to its specific target. On the other hand, the second peak reduces the surface heat wave. The suggested MPA (Ag@ZnO) structure is activated by a plane wave using a full wave vector and a broad frequency domain solution. In the framework of computer simulation technology (CST) Microwave Studio, uses both Finite-Difference-Time-Domain (FDTD) and Finite-Element-Method (FEM) techniques to predict the optical behavior of the proposed MPA structure. Both peaks achieved a high value of absorptivity due to the simultaneous excitation of the electric and magnetic dipole at resonance wavelength.

## Introduction

Infrared (IR) stealth technology is a component of military design dedicated to reducing the infrared signature of objects, making them less detectable^1–3^. To reduce the heat emissions that infrared sensors can detect, certain materials and combinations are employed. IR stealth is mostly used in military aircraft, helicopters, land vehicles, battleships, and individual soldiers^4–7^. It reduces the risk of investigation, which improves operational security. In defence-related scenarios, it is crucial for improving mission effectiveness and survivability. On the other hand, IR perfect absorbers can be utilized in various today’s technologies like daytime passive relative cooling, thermal management in electronics, medical imaging, and enhanced IR sensors^8^.

As a result of the development of infrared technology, all infrared signals emitted or reflected by objects, including those from laser-guided devices, may now be detected due to more advanced detection systems and accurate tracking technology^9,10^. To reduce the amount of heat that surfaces emit and to minimize the scattering or reflecting properties of radar waves, efforts have been made. The temperature and wavelength of the radiation are the main factors that affect how much-infrared light is attenuated when it passes through the atmosphere. Many types of atmospheric gases, such as CO_2_, H_2_O (vapors), O_3_, CO, and others, absorb and deflect infrared radiation^11–13^. These specific ranges of 1–14 µm of bands in the atmospheric windows have good IR transparency, regardless of the atmosphere's ability to absorb IR radiation. For this reason, these bands are employed for detection. Outside the atmospheric windows, there is a significant attenuation of infrared radiation because of the absorption and scattering of CO_2_ and H_2_O (vapor)^14–18^.

Perfect optical absorbers are an essential component of stealth technology, which is used in many different applications. Plasmonic-based metal-insulator-metal metamaterial perfect absorbers have attracted a lot of attention, as extremely promising and well-researched components that can function over a broad range of wavelengths^19–21^. They have low emissivity, high absorbance, and efficiency at different incident angles, which all contribute to their outstanding performance. Metamaterial Perfect Absorbers (MPA) have been successfully applied in the field of stealth technology to reduce the cross-section of electromagnetic waves in the GHz to THz range^22–25^. They can function selectively at particular frequencies, which are explained by their remarkable absorption capacities. In an earlier study, a metal-insulator-metal structure comprising a silver (Ag) disc on a substrate containing metal and dielectric layers was developed. In the designed Zinc Oxide embedded Silver structure, Ag is chosen for its high conductivity, optimizing efficient SPP excitation, while ZnO, with its high refractive index, confines SPPs at the metal-dielectric interface, enhancing field intensity and absorption efficiency. However, silver can undergo oxidation quickly in the presence of air, which makes it non-plasmonic. To avoid oxidation and preserve the plasmonic characteristics, we used a Zinc Oxide embedded Silver (Ag@ZnO) MPA structure in our present design.

We used the FDTD and FEM methods to predict the optical response of the design (Ag@ZnO) MPA structure in the framework of CST Microwave Studio^26,27^. The proposed (Ag@ZnO) metamaterial structure was excited by plane waves. The optical response, including parameters like transmittance, scattering, and absorbance as well as the far- and near-field optical behaviors of the proposed metamaterial structures, is predicted by the CST software by efficiently solving the Maxwell equations^28,29^.

## Simulation, design, and dimensions of proposed structure

**Fig. 1:**
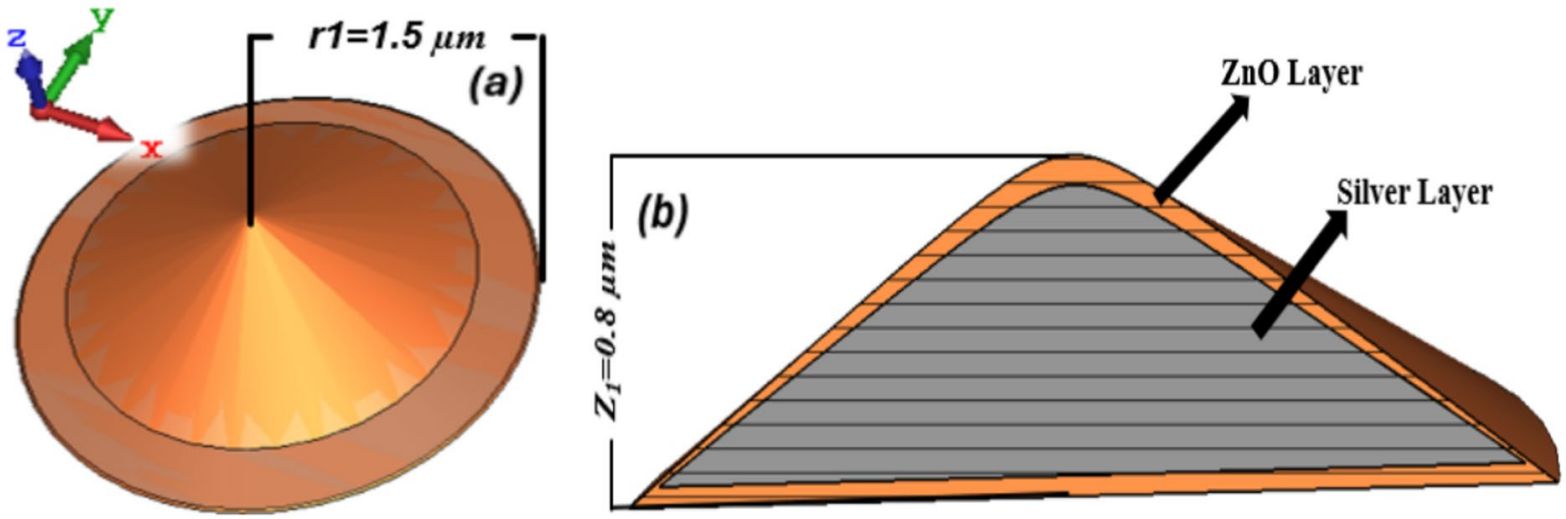
(**a**) Conical shape of zinc oxide embedded Ag MPA structure having a bottom radius (*r*_1_ = 1.5) (**b**) cross-sectional view of the zinc oxide layer and a silver layer of the MPA structure.

The cone-shaped MPA structure is a good absorber due to continuous change in its cross-section area, which can support a wider spectrum of electromagnetic wave absorption^30^. The designed new selective plasmonic-based Metamaterial Perfect Absorber (MPA) consists of zinc oxide embedded silver (Ag@ZnO), to achieve absorptivity/emissivity in the infrared spectrum spanning from (1–14 µm). Figure 1a shows the cone-shaped MPA structure. It is composed of zinc oxide (ZnO) as the dielectric material and silver (Ag) metal with inherent losses. A 1.5 µm bottom radius (*r*_1_) and a 0 µm top radius (*r*_2_) represent the cone's dimensions. Along the negative *Z*-axis, the design (Ag@ZnO) structure is excited by an incoming plane wave that strikes the surface of the metamaterial. Within the *YZ* plane, the electric field has zero magnitude and is aligned along the positive *X*-axis. Concurrently, the magnetic field aligns with the positive *Y*-axis and is perpendicular to the direction that the incident light is propagating. In the *XY* plane, its magnitude is zero. The proposed MPA structure optical response is examined for incident light in the 1–1000 THz frequency range. Figure 1b shows the cross-sectional view of the design MPA structure having silver and zinc oxide layers, the height of the cone is 0.8 µm which is represented by *Z*_1_. Dual absorption peaks of the design MPA Ag@ZnO structure are visible at different wavelengths in the range of (1–3 µm) and (5–8 µm). The equation $A = 1 - R$ can be employed to determine the absorption (*A*) through the reflection (*R*) since the metal layer is sufficiently broad to completely block the transmission of light^31^. This structure is discretized to optimize it into smaller meshes for more efficient numerical evaluations.

Within the context of the CST microwave studio, the optical behavior of the design Ag@ZnO MPA structure has been predicted using FDTD and FEM techniques. The proposed MPA structure was excited with plane waves, and its predicted optical properties were evaluated in a wide frequency range spanning from 1 to 1000 THz. Using the FDTD approach, Maxwell's equations have been numerically solved in both the temporal and spatial domains^28^. FDTD is based on the concept of differentiating the electric and magnetic fields in space and time. For a one-dimensional cone, we could include the radial geometry in the standard FDTD update equations. For a one-dimensional cone, the FDTD update equations are shown below.

Update Equation for Electric Field (*E*_*r*_)

$$ E_{r}^{k + 1} \left( n \right) = E_{r}^{k} \left( n \right) + \frac{\Delta t}{{\epsilon_{r} \left( n \right)}}\left( {\frac{1}{r}\frac{d}{dr}\left( {rH_{\theta }^{k} \left( n \right)} \right) - J_{r}^{k} \left( n \right)} \right) $$

Where $E_{r}$ is the radial electric field component, $\epsilon_{r}$ is the radial permittivity, from the above equation $H_{\theta }$ represents azimuthal magnetic field component, and “$J_{r}$” represents the radial current density, where “*n*” represents the grid point index, and “*k*” is the time step.

Update Equation for Magnetic Field $\left( {H_{\theta } } \right)$

$$ H_{\theta }^{k + 1} \left( {n + \frac{1}{2}} \right) = H_{\theta }^{k} \left( {n + \frac{1}{2}} \right) + \frac{\Delta t}{{\mu_{r} \left( {n + \frac{1}{2}} \right)}}\left( {\frac{1}{{r + \frac{\Delta r}{2}}}\frac{d}{dr}\left( {rE_{r}^{k + 1} \left( {n + 1} \right)} \right)} \right) $$

where $H_{\theta }$ is the azimuthal magnetic field component, $\mu_{r}$ is the radial permeability, and $\Delta r$ is the radial grid spacing.

To account for the radial components of the electric and magnetic fields relevant to a one-dimensional cone, the presented equations are a modification of the usual FDTD equations designed for a cylindrical coordinate system. In order to model the electromagnetic wave propagation in the cone structure, the radial derivatives are estimated using central difference techniques, and the update equations are applied repeatedly in both time and space^32^. Depending on the nature of the problem and the required accuracy, the precise implementation details might need to be changed.

## Result and discussions

**Fig. 2:**
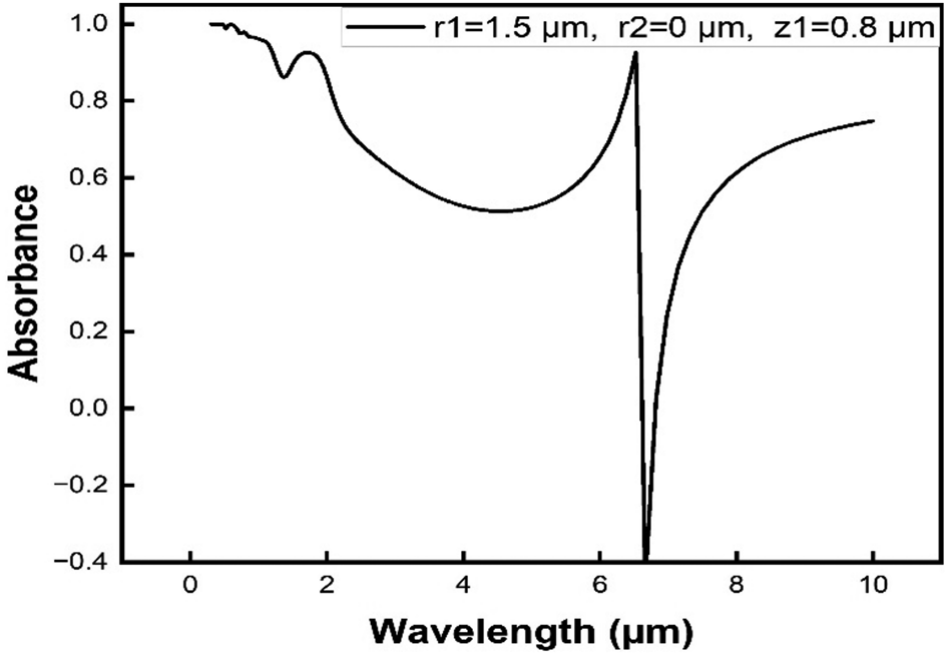
Wavelength-dependent absorption spectra of the designed Ag@ZnO MPA structure.

The spectral characteristics of the design Ag@ZnO MPA structure are shown in Fig. 2 with a solid line. This figure shows the absorptivity/emissivity of the proposed geometrical model consisting of Zinc Oxide embedded silver MPA structure within the infrared domain spanning from 1 to 14 µm. An instance where a plane wave with p-polarization impinges perpendicularly onto the metamaterial's surface is taken into consideration^33^. The wavelength of the incident light, expressed in micrometers, is plotted along the *X*-axis in Fig. 2, while the amount of light absorbed is plotted along the *Y*-axis. The optical behavior of the designed MPA structure has been examined by numerical simulations employing the FDTD technique. The result included determining precisely the Surface Plasmon Polariton (SSP) wavelength, which is defined as “electromagnetic waves traveling along the dielectric or metal-air interface, typically in the visible or infrared frequency range”. Furthermore, we determined the wavelength corresponding to the Magnetic Polariton (MP), which is characterized as “the strong coupling of electromagnetic waves with electric or magnetic dipoles that results in an elementary excitation in materials”^9,34^. Two different perfect absorption peaks that occur at various wavelengths and positions are shown in Fig. 2. Peak *A* is the name given to the first peak, and Peak *B* to the second one*.* Peak *A* significantly decreases the scattering of infrared laser beams from surfaces at a wavelength of 1.7 µm with an absorptivity of ≈ 93.1%. In order to suppress IR signatures that prevent laser-guided devices from focusing on their targets, this reduction in scattering is essential^35,36^. Furthermore, Peak *B* has a high absorptivity of ≈ 93.5% while measured at a wavelength of 6.5 µm. This specific peak reduces the amount of heat waves that surfaces emit. *Z*_1_ (height) determines the resonance wavelength of the SSP wave, which is fixed at 0.8 µm. This particular arrangement is chosen to satisfy the necessary conditions for IR stealth technology and reduce the scattering issues associated with peak *A* laser light emitted from surfaces. To generate an additional MP resonance peak at 6.5 µm, we modified the design structure's dimensions. The dual-band zinc oxide embedded silver MPA structure has remarkably low emissivity as well as excellent absorptivity in the near-wavelength infrared (NWIR) and mid-wavelength infrared (MWIR) wavelength bands^37,38^. This feature makes it possible to reduce the amount of heat emission, which reduces the amount of radiation that may be detected by various infrared tracking and imaging devices.

**Fig. 3:**
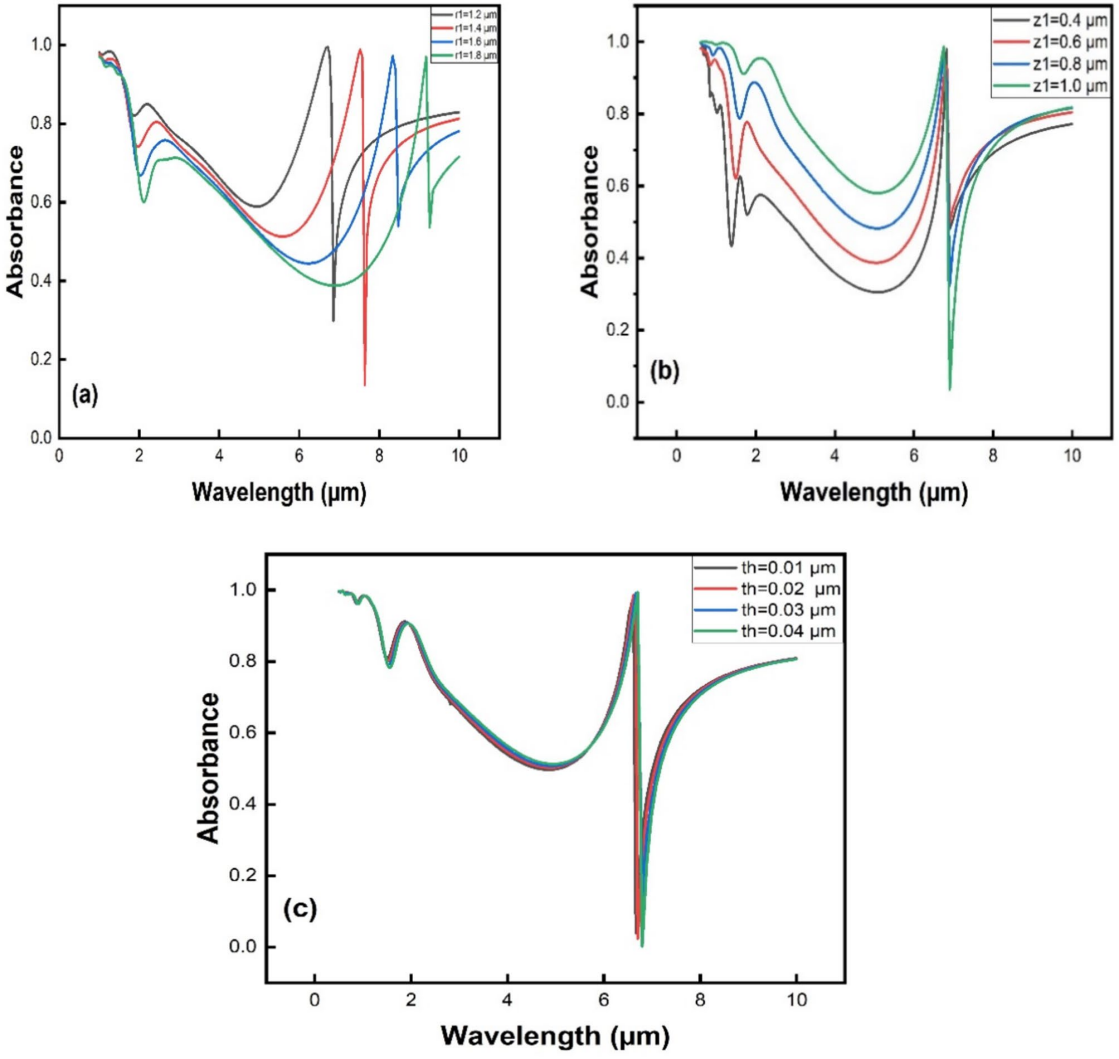
Absorption intensities for various structural parameters (**a**) absorption intensities at various bottom radius values (**b**) absorption intensities at various heights of the cone (**c**) absorption intensities at various thicknesses of the cone.

In the proposed MPA structure, Fig. 3 shows the relationship between the absorption peak and variations in structural parameters, especially the height of the cone, thickness of the cone, and bottom radius of the cone*.* However, there is an obvious red shift in the absorption peak when the bottom radius of the cone is increased as shown in Fig. 3a. The absorption peak at resonance wavelength shifts from 6.7 to 8.5 µm, which leads to a high absorptivity when the bottom radius varies between 1.2 µm and 1.6 µm while keeping other parameters like the cone height (*Z*_1_ = 0.8 µm) and cone thickness (0.01 µm) constant. Figure 3b shows that the absorption intensity is highly affected by the variation in the cone's height, denoted by *Z*_1_. The absorptivity at 1.3 µm increases from 42.5 to 60% when the cone height increases from 0.4 to 0.6 µm while keeping other parameters constant, such as the bottom radius (*r*_1_ = 1.5 µm) and cone thickness (0.01 µm). The absorption intensity at 1.3 µm experiences a further increase, reaching ≈ 93.1% when *Z*_1_ is set to 1.0 µm. Peak *A* and Peak *B* absorption intensities at the nanoscale are unaffected by variations in the ZnO layer's thickness, as seen in Fig. 3c. Moreover, the IR absorption behavior of the array of the proposed Ag@ZnO structure strongly depends on the distance between the unit structures and has been discussed in Supplementary Materials.

**Fig. 4:**
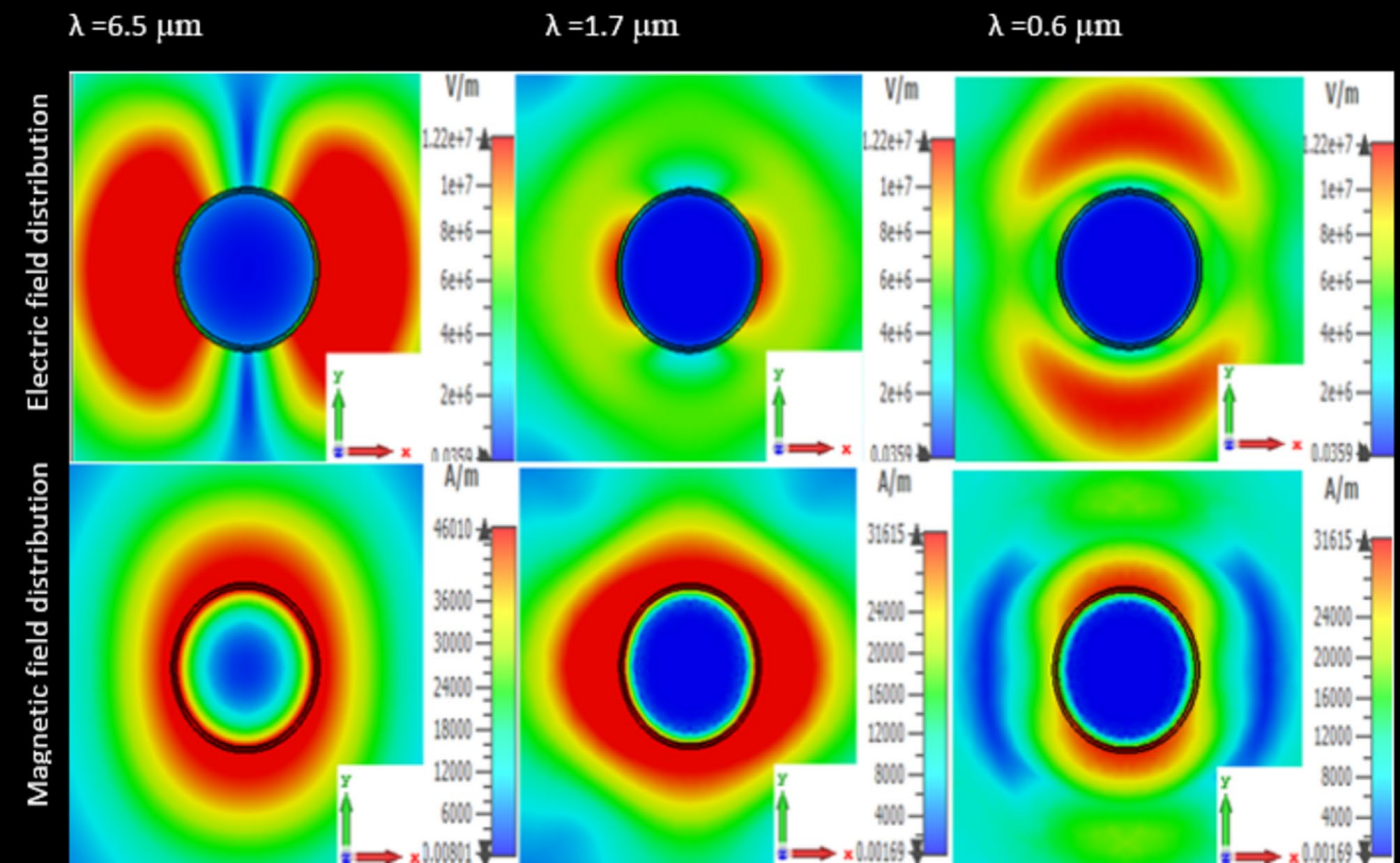
Distribution of electric and magnetic field inside MPA structure for different resonance wavelengths.

Figure 4 represents the electric and magnetic polarization inside the Ag@ZnO structure that results from the excitation of a plane wave in the cross-section of the proposed metamaterial structure at the resonance wavelengths of SPP and MP^39,40^. Figure 4 shows that the activation of an electric dipole within the proposed metamaterial structure is indicated by the electric field distribution. The simulation results also show that the electric field is concentrated locally inside the metamaterial structure. From Fig. 4 at the resonance wavelength of *λ* = 6.5 µm, the electric dipole mode inside the MPA structure which shows opposite polarization charges but equal magnitudes along the *X*-axis is essential to maintain high absorptivity^41–43^. More specifically, phase angles *ϕ* = 90° and *ϕ* = 270° are obtained at this resonance wavelength. Both the excitation of an electric dipole and a high concentration of the electric field arise simultaneously at the resonance wavelength of *λ* = 1.7 µm, with phase angles of *ϕ* = 130 ° and *ϕ* = 280 °. At the resonance wavelength (*λ* = 0.6 µm)*,* similar phenomena are observed with corresponding phase angles of *ϕ* = 22 ° and *ϕ* = 202.5 °. Figure 4 shows that inside the Ag@ZnO MPA structure at all three resonance wavelengths the excitation of magnetic dipoles and the high concentration of the magnetic field distribution due to incident plane wave^44^, each with a distinct phase angle. The simulation results clearly show that the maximum absorption peaks observed at resonance wavelengths (*λ* = 6.5 µm, *λ* = 1.7 µm, and *λ* = 0.6 µm)^45,46^ and their corresponding phase angles (*ϕ* = 11.5 ° and *ϕ* = 247 °, *ϕ* = 22 ° and *ϕ* = 270 °, *ϕ* = 45 ° and *ϕ* = 326 °) are directly caused by the distribution of magnetic fields and the activation of the magnetic dipole inside the MPA structure^47^.

**Fig. 5:**
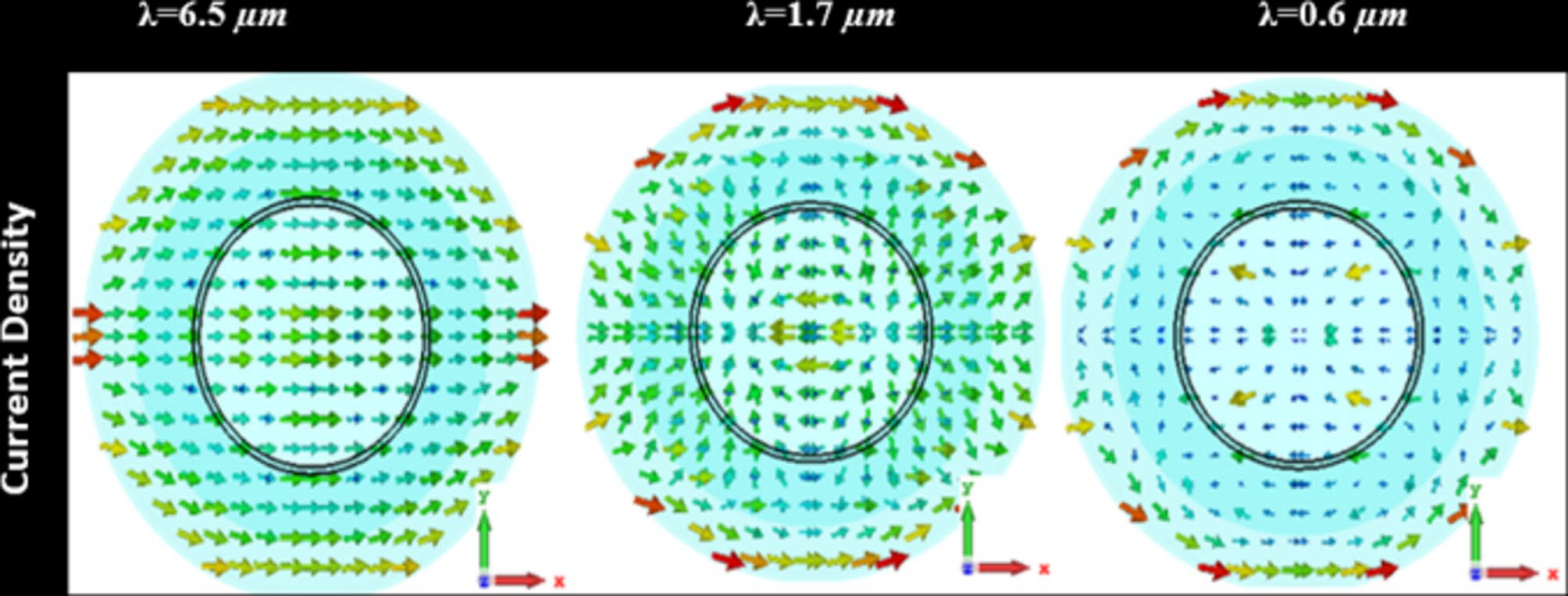
Distribution of current density inside MPA structure at resonance wavelength.

An electromagnetic wave, normally incident along the negative *Z*-axis, is applied to a structure made of Zinc oxide-embedded Silver (Ag@ZnO)^48^. The metallic component experiences a current density as a result of this interaction, which affects the absorption characteristics. The absorption peaks at resonance wavelengths occur due to the simultaneous excitation of electric dipoles along the *X*-axis and magnetic dipoles along the *Y*-axis. The current density pattern within the Ag@ZnO structure is depicted in Fig. 5. Driven by the oscillating electric field, the incident electromagnetic wave causes surface currents to flow through the metal. The rate of current density is directly related to this absorbed energy^49,50^. In response to the incident field, the dielectric component simultaneously experiences polarization and exhibits dielectric absorption, which temporarily stores and releases electrical energy. The current density in the metal shapes the absorption properties of the dielectric and affects the strength of the electric field inside the dielectric^51,52^. Based on the FDTD simulation results, as can be seen in Fig. 5, high absorption is induced by current density inside the MPA structure at resonance wavelengths (*λ* = 6.5 µm, *λ* = 1.7 µm, and *λ* = 0.6 µm).

## Conclusion

We have introduced a dual-band MPA (Ag@ZnO) structure to maintain invisibility in the presence of laser radar targeting wavelengths at 1.7 µm and 6.5 µm. When electromagnetic waves strike the surface of metamaterial along the negative Z-axis, the proposed (Ag@ZnO) structure shows outstanding performance across a broad frequency range, spanning from (1–1000 THz). It achieves absorptivity levels of approximately 93.1% and 93.5% at the respective target wavelengths. The suggested structure composed of Zinc Oxide embedded Silver has the potential to achieve laser stealth capabilities by minimizing the infrared signature detectable by laser-guided devices. This is due to its ability to absorb the amount of incident electromagnetic waves along the negative *z*-direction. By employing the multiple resonant peak superposition method, we can effectively increase the absorption bandwidth from 0.1 to 3 µm. Based on the simulation results, the absorptivity/emissivity in the (1.2–2 µm) region is more than 80%. The physical mechanism behind absorption peaks at resonance wavelengths of 1.7 µm and 6.5 µm is explained by the excitation of electric and magnetic fields along the *X* and *Y*-axis, which are caused by electric and magnetic dipoles. A numerical analysis is presented to assess the suitability of the proposed MPA (Ag@ZnO) structure for IR stealth technology.

## Supplementary Information

Supplementary Information.

## Data Availability

Data is available throughout the manuscript and supporting files.
