# Supplementary material for: Selective-wavelength perfect infrared absorption in Ag@ZnO conical metamaterial structure
Source: Sci Rep. 2024 Sep 12;14:21321. doi: 10.1038/s41598-024-71260-2 (PMC11393131; doi:10.1038/s41598-024-71260-2)
Supplement: Supplementary file 1 — Supplementary Information. [file 41598_2024_71260_MOESM1_ESM.docx]

**Supplementary material**

1. **IR Absorption of the array of the proposed metamaterial structure**

A periodic metamaterial structure with two successive cones of zinc oxide embedded silver is shown in *Supplementary Fig.* The objective of the proposed structure is to effectively absorb incident light in the wide frequency range of *1 THz to 1000 THz*. The successive cone design's tunability is one of its main benefits. Cones' dimensions and material composition can be precisely tuned to focus on particular light frequencies, providing a tailored solution that meets the requirements of infrared *(IR)* stealth technology. Because of its tunability, the metamaterial may be optimized to achieve certain absorption properties, which improves its stealth technology performance by reducing its infrared signature. Two cone-shaped layers are arranged one after the other to form the periodic structure. The geometric parameters of each cone are as follows: the height of the cone *(Z1)* is *0.8 µm*, the top radius *(r2)* is *0 µm,* the bottom radius *(r1)* is set to *1.5 µm*, and the spacing *(d)* between two consecutive cones is *0.1 µm*. All cones in the periodic structure have the same dimensions, which is significant. With this configuration, the metamaterial's electromagnetic characteristics may be precisely controlled, making it easier to adjust incident light across a wide frequency range for applications such as infrared stealth technology.


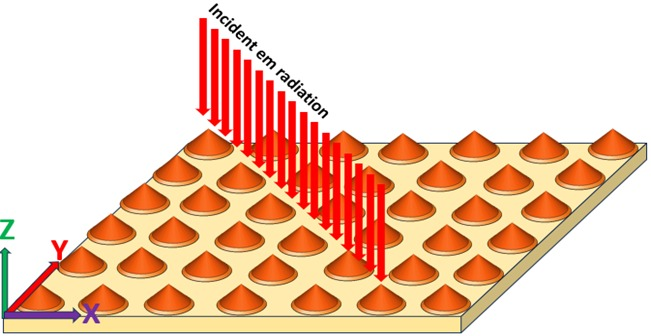


*Supplementary Fig.1: Periodic behaviour of Conical shape of Zinc Oxide embedded Ag MPA structure having a bottom radius (r1=1.5) of both cones and the distance between two consecutive d=0.1 µm.*

The relationship between the absorption intensities as a function of wavelength and the structural parameters such as the bottom radius of the cones and the spacing between two consecutive cones is depicted in *Supplementary Fig. 2(a)* in particular shows how variations in the bottom radius of the cones' affect the absorbance peaks. The wavelength of the absorption peak is observed to redshift from *6.7 µm* to *9.8 µm* when the bottom radius of the cone is increased from *0.7 µm* to *1.0 µm*, but the distance between subsequent cones remains constant. Furthermore, *Supplementary Fig.2(b) & 2(c)* show the relationship between changes in the distance between two adjacent cones and the corresponding absorption peaks. *Supplementary Fig.2(b)* shows that absorption peaks occur within the infrared domain when the distance between two successive cones is minimized. On the other hand, as *Supplementary Fig.2(c)* shows, the absorption maxima move away from the infrared domain when the cones are positioned farther apart, i.e., the distance between them is increased. The significance of precise control over structural parameters in focusing on the absorption characteristics for applications such as infrared stealth technology is shown by this observation, which shows the sensitivity of the absorption properties to changes in the spatial arrangement of the metamaterial structure.


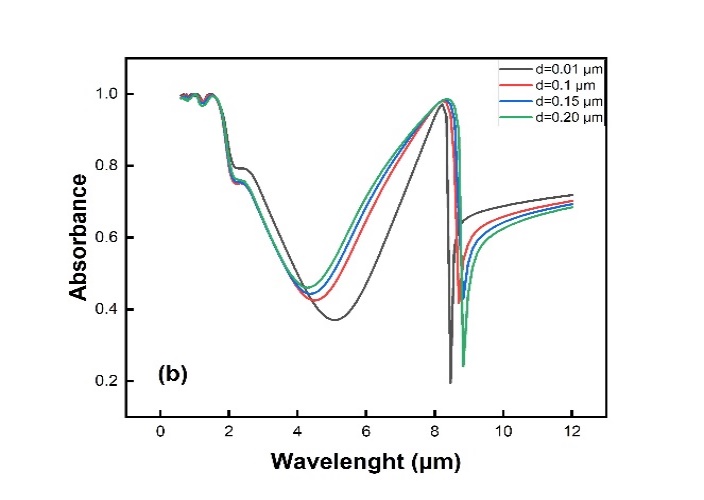

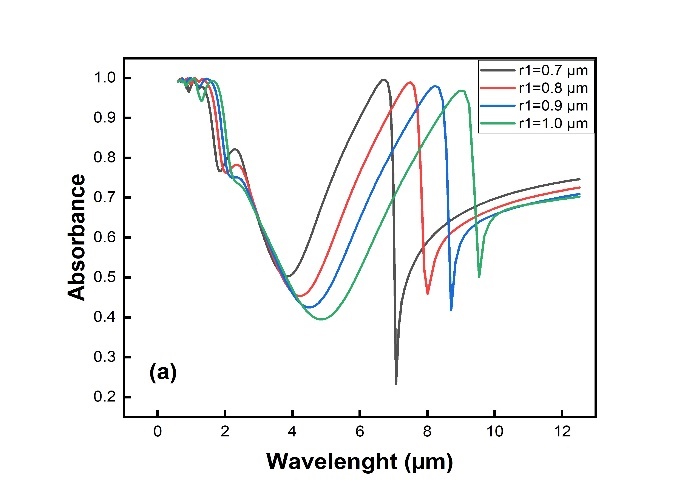


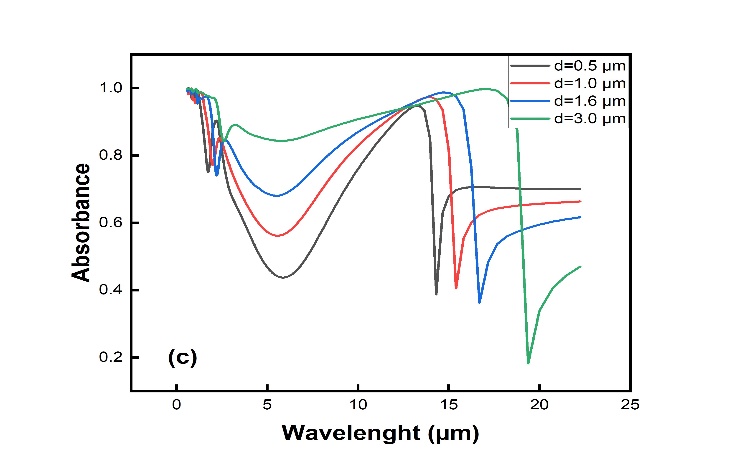


*Supplementary Fig.2(a-c): Absorption spectra as a function of wavelength for different structural perimeter (a) Absorption intensities at various bottom radii (b) Absorption intensities for a minimum distance between two consecutive cones (c) Absorption intensities for a maximum distance between two consecutive cones.*

1. **Far Field Directivity of the single proposed structure**

The Far-Field directivity properties of the proposed Metamaterial Perfect Absorber (MPA) radiating structure, which is made of Zinc Oxide embedded Silver *(Ag@ZnO),* are shown in *Supplementary Fig.3.* In this configuration, the electric and magnetic fields align along the positive *X-axis* and positive *Y-axes,* respectively, and the *MPA* radiating structure is excited by a vertically incident plane wave along the negative *Z-axis*. At different resonance wavelengths *(λ=6.5 µm, λ=1.7 µm, and λ=0.6 µm),* *Supplementary Fig.3* shows the Far Field Directivity. The intensity of infrared radiation emitted or reflected in the primary direction of interest is determined by the magnitude of the main lobe, as seen in *Fig.3*. The main lobes' magnitudes are measured to be *(6.94 dBi, 7.89 dBi,* and *16.9 dBi)* respectively, at various resonance wavelengths *(6.5 µm, 1.7 µm,* and *0.6 µm).* Reducing the magnitude of the main lobe is an important way to reduce an object's detectability in the context of infrared *(IR)* stealth technology and also lowers the object's infrared signature. The main lobe direction in *Fig.3* is marked at *180*°, indicating that all electromagnetic radiation scatters in the forward direction. Notably, the proposed *MPA* radiating structure exhibits zero backward scattering at all three resonance wavelengths. *Supplementary Fig.3* emphasizes the importance of controlling the magnitude and direction of the main lobe to minimize an object's infrared signature and improve its survivability against detection and targeting by infrared sensors in IR stealth technology.

*
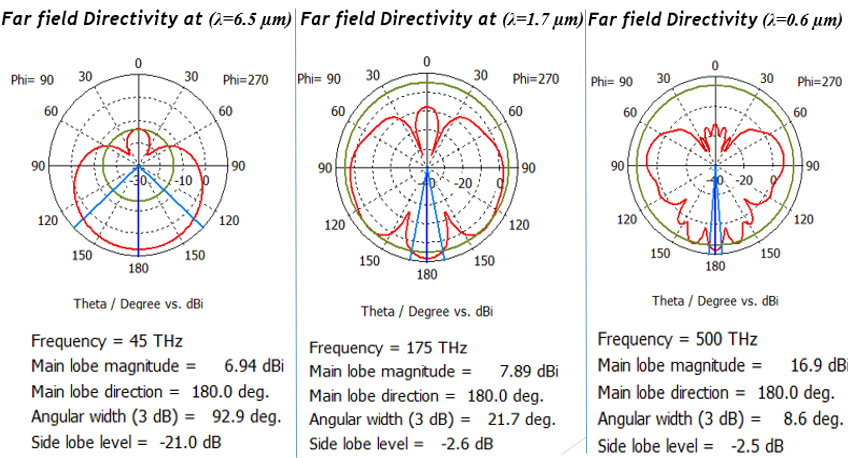
*

*Supplementary Fig.3: Far Field Directivity of the proposed design MPA structure at different resonance wavelength*
